# Prior exercise in humans redistributes intramuscular GLUT4 and enhances insulin-stimulated sarcolemmal and endosomal GLUT4 translocation

**DOI:** 10.1016/j.molmet.2020.100998

**Published:** 2020-04-17

**Authors:** Jonas R. Knudsen, Dorte E. Steenberg, Janne R. Hingst, Lorna R. Hodgson, Carlos Henriquez-Olguin, Zhencheng Li, Bente Kiens, Erik A. Richter, Jørgen F.P. Wojtaszewski, Paul Verkade, Thomas E. Jensen

**Affiliations:** 1 Molecular Physiology Section, Department of Nutrition, Exercise and Sports, Faculty of Science, University of Copenhagen, August Krogh Building, Universitetsparken 13, 2100, Copenhagen Oe, Denmark; 2 Laboratory of Microsystems 2, Institute of Microengineering, Ecole Polytechnique Fédérale de Lausanne, Batiment BM, 1015, Lausanne, Switzerland; 3 School of Biochemistry, University of Bristol, Biomedical Sciences Building, University Walk, BS8 1TD, Bristol, United Kingdom

**Keywords:** Exercise, Skeletal muscle, GLUT4, Insulin sensitivity, Insulin-resistance

## Abstract

**Objective:**

Exercise is a cornerstone in the management of skeletal muscle insulin-resistance. A well-established benefit of a single bout of exercise is increased insulin sensitivity for hours post-exercise in the previously exercised musculature. Although rodent studies suggest that the insulin-sensitization phenomenon involves enhanced insulin-stimulated GLUT4 cell surface translocation and might involve intramuscular redistribution of GLUT4, the conservation to humans is unknown.

**Methods:**

Healthy young males underwent an insulin-sensitizing one-legged kicking exercise bout for 1 h followed by fatigue bouts to exhaustion. Muscle biopsies were obtained 4 h post-exercise before and after a 2-hour hyperinsulinemic-euglycemic clamp.

**Results:**

A detailed microscopy-based analysis of GLUT4 distribution within seven different myocellular compartments revealed that prior exercise increased GLUT4 localization in insulin-responsive storage vesicles and T-tubuli. Furthermore, insulin-stimulated GLUT4 localization was augmented at the sarcolemma and in the endosomal compartments.

**Conclusions:**

An intracellular redistribution of GLUT4 post-exercise is proposed as a molecular mechanism contributing to the insulin-sensitizing effect of prior exercise in human skeletal muscle.

## Highlights

• Intramyocellular GLUT4 is redistributed 4 h after exercise in humans.
• GLUT4 content is increased in GLUT4 storage vesicles and T-tubuli post-exercise.
• Prior exercise + insulin increases sarcolemmal and endosomal GLUT4.
• GLUT4 redistribution may thus contribute to post-exercise muscle insulin-sensitization.

## Graphical abstract

**Figure:**
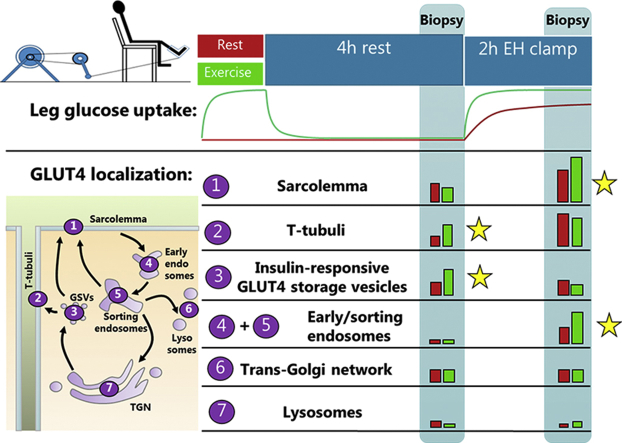

## 1 Introduction

Exercise is a key health-promoting activity and a cornerstone in the prevention and treatment of insulin-resistance in skeletal muscle. A known benefit of each bout of exercise is its ability to acutely increase insulin sensitivity, understood here as an enhanced ability of a submaximal dose of insulin to promote muscle glucose uptake. This increased insulin sensitivity is consistently observed for many hours post-exercise in the previously exercised musculature compared to resting muscle. Molecularly, rodent studies suggest that augmented insulin-stimulated recruitment of intra-myocellular glucose transporter 4 (GLUT4) proteins to the cell surface, to facilitate glucose entry, plays a crucial role in this insulin-sensitization phenomenon [1,2].

The research field has, since the first description of the insulin-sensitization phenomenon in rats [3] and humans [[4], [5], [6]] in the 1980s, mostly sought a molecular explanation for insulin sensitization at the cell signaling level, specifically within the insulin-IRS-PI3K-Akt-TBC1D4 pathway suggested to regulate insulin-stimulated GLUT4 translocation in cell culture and rodents. Human studies have not demonstrated differences in the insulin-stimulated activation of the proximal insulin signaling cascade post-exercise [7,8] but have more recently observed exercise-induced changes in the phosphorylation of the GLUT4 translocation-linked Akt substrate TBC1D4 3–4 h post-exercise [9], which might contribute to insulin-sensitization. However, the downstream signaling consequences of exercise-induced TBC1D4 phosphorylation changes remain unknown. More critically, insulin-sensitization of GLUT4 translocation to the surface membrane by prior exercise has not been demonstrated in humans, making it uncertain whether this process contributes to insulin-sensitization in human skeletal muscle.

A fundamentally different hypothesis explaining augmented insulin-stimulated GLUT4 translocation post-exercise is that the “memory” of prior exercise is retained in the intracellular localization of GLUT4 and not necessarily at the cell signaling level. This is an attractive hypothesis, since other vesicle translocation-based processes in non-muscle cell types, e.g., neurotransmitter vesicles in neurons and insulin-containing vesicles in pancreatic β-cells, increase the number of vesicles available for exocytosis after a period of stimulation [10,11]. Interestingly, a rat study by John Holloszy's laboratory in 2006 [2] suggested that prior exercise might redistribute GLUT4 intracellularly to allow the same insulin-signal to recruit more GLUT4 to the cell surface, the latter confirming their own original observations [1]. However, this proposal was based on measurements of muscle glucose uptake and surface membrane GLUT4 in isolated incubated rat muscles and the direct investigation of intramyocellular redistribution of GLUT4 post-contraction, which is required to formally test this hypothesis, has not been performed in any species.

In the current study, we performed a comprehensive microscopy-based analysis of GLUT4 distribution throughout the endomembrane system in human muscle biopsies obtained 4 h after a single bout of one-legged kicking exercise before and after a 2-hour hyperinsulinemic-euglycemic clamp. This model has repeatedly demonstrated markedly increased insulin-stimulated glucose uptake in the exercised compared to the rested legs [5,8,[12], [13], [14], [15]], and we used it to directly test 1) if intramyocellular GLUT4 was redistributed by prior exercise and 2) if prior exercise enhanced insulin-stimulated GLUT4 translocation to the sarcolemma and T-tubule surface membranes in human skeletal muscle. We show for the first time in humans that insulin-sensitizing exercise increases GLUT4 within insulin-responsive GLUT4 storage vesicles (GSVs) and T-tubuli post-exercise prior to insulin-stimulation. Furthermore, we observed enhanced insulin-stimulated GLUT4 accumulation in the sarcolemma and endosomal compartments. We interpret this as a molecular signature induced by a single bout of exercise contributing to the post-exercise insulin-sensitization phenomenon.

## 2 Results

### 2.1 Sample thinning enhanced resolution microscopy (STERM) allows immunofluorescence co-localization microscopy with markedly improved Z-plane resolution

**Figure 1:**
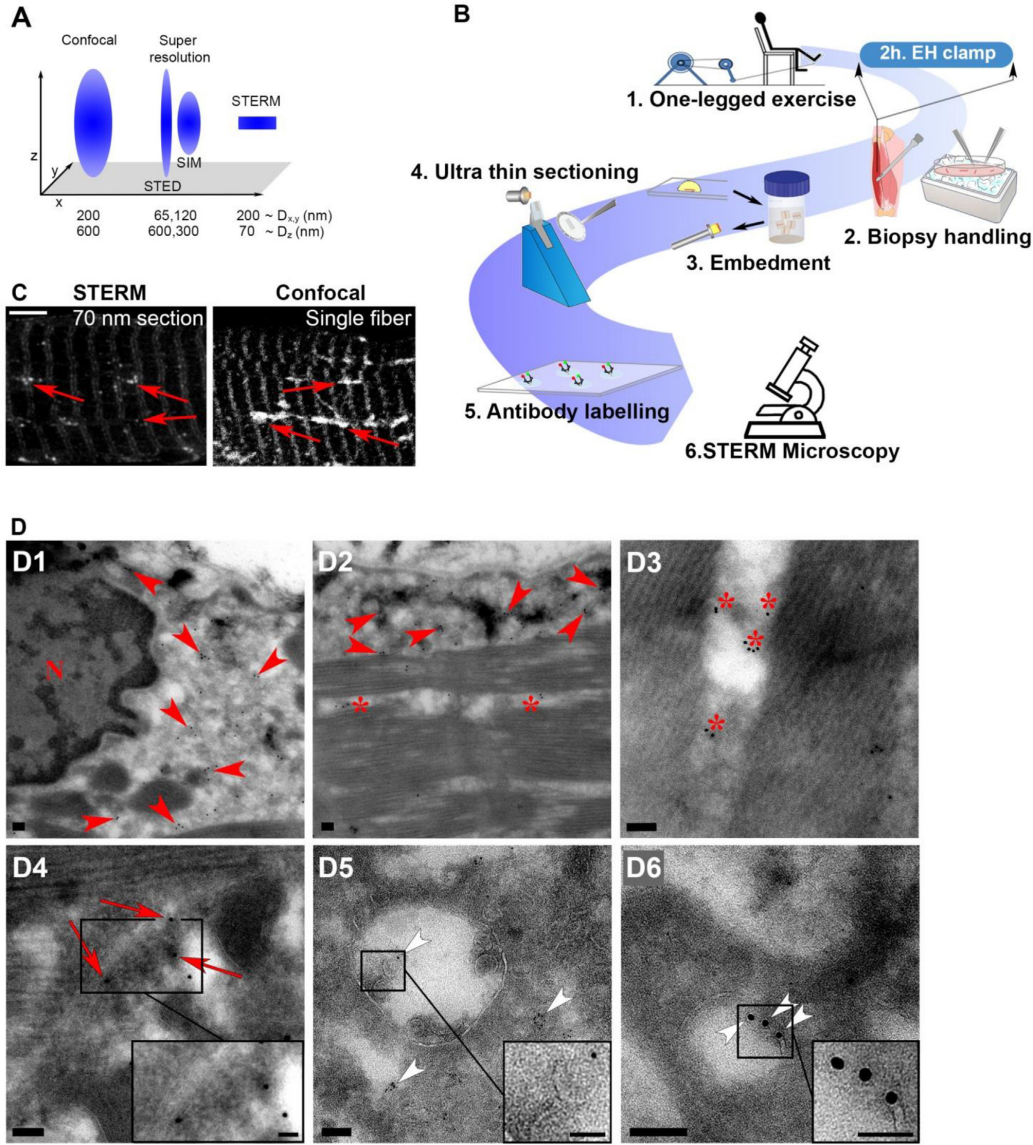
**Validation of a simple method for high-resolution imaging of endogenous GLUT4.** (A) Theoretical resolvable volume for confocal microscopy, stimulated emission depletion (STED) and structured illumination super resolution microscopy (SIM) and the sample thinning enhanced resolution microscopy (STERM) used in the current study. D = diameter. It should be noted that these theoretical resolution values are estimates of the actual resolution of the techniques, since several factors affect the final imaging resolution. Figure modified from [16]. (B) Workflow overview for STERM. (1) Subjects underwent kicking exercise until exhaustion and (2) muscle biopsies were obtained and fixed before and after a hyperinsulinemic-euglycemic clamp. (3) Bundles of fibers were embedded in gelatin, sucrose infiltrated and mounted on cryo-pins. (4) Ultra-thin 70 nm sections were generated and (5) GLUT4 and subcellular compartment markers were labelled and (6) imaged. (C) Comparison of resolution by images obtained by confocal imaging of whole single mouse muscle fibers and 70 nm STERM sections prepared as illustrated in A. Red arrows mark vesicles which were better resolved by STERM. (D) Micrographs from transmission electron microscopy of human muscle showing GLUT4 in the perinuclear region (D1), the subsarcolemmal region (D2), the intrafibrillar region (D3), in tubule-vesicular regions (D4), in multi-vesicular endosomes (D5) and in individual vesicles (D6). Red arrowheads mark perinuclear and subsarcolemmal GLUT4, asterisks mark intrafibrillar GLUT4, red arrows mark GLUT4 in tubulovesicular structures and white arrowheads mark GLUT4 in vesicles. Scale bar = 5 μm (C) and 100 nm (D).

Imaging the overlap between immuno-labelled proteins of interest by light microscopy, mainly done using Confocal Laser Scanning Microscopy, is one of the most widely used techniques used to infer subcellular co-localization in biological systems. The resolution in the planes of imaging, i.e., laterally (X/Y) and axially (Z), determines the resolving power of such co-localization. The focal X/Y plane resolution in fluorescence microscopy has been improved well below the diffraction barrier from ∼200 nm by confocal microscopy to ∼65 nm by some super-resolution techniques. In contrast, the axial resolution is ∼600 nm with standard confocal microscopy and remains ∼300 nm or above with individual super-resolution techniques, discounting techniques such as total internal reflection fluorescence (TIRF), restricted to imaging the 150–200 nm closest to the cover-slip (Figure 1A) [16]. This is a major limitation, especially when imaging deep tissue samples, since poor axial resolution will in practice decrease the X/Y resolution and furthermore increase the likelihood of false-positive co-localization of distant fluorophores in the Z-plane. In addition, permeabilization using detergents is commonly used to allow intracellular antibody penetration into the tissue sample, but this may cause wash-out of smaller membrane-compartments [17].

**Figure 2:**
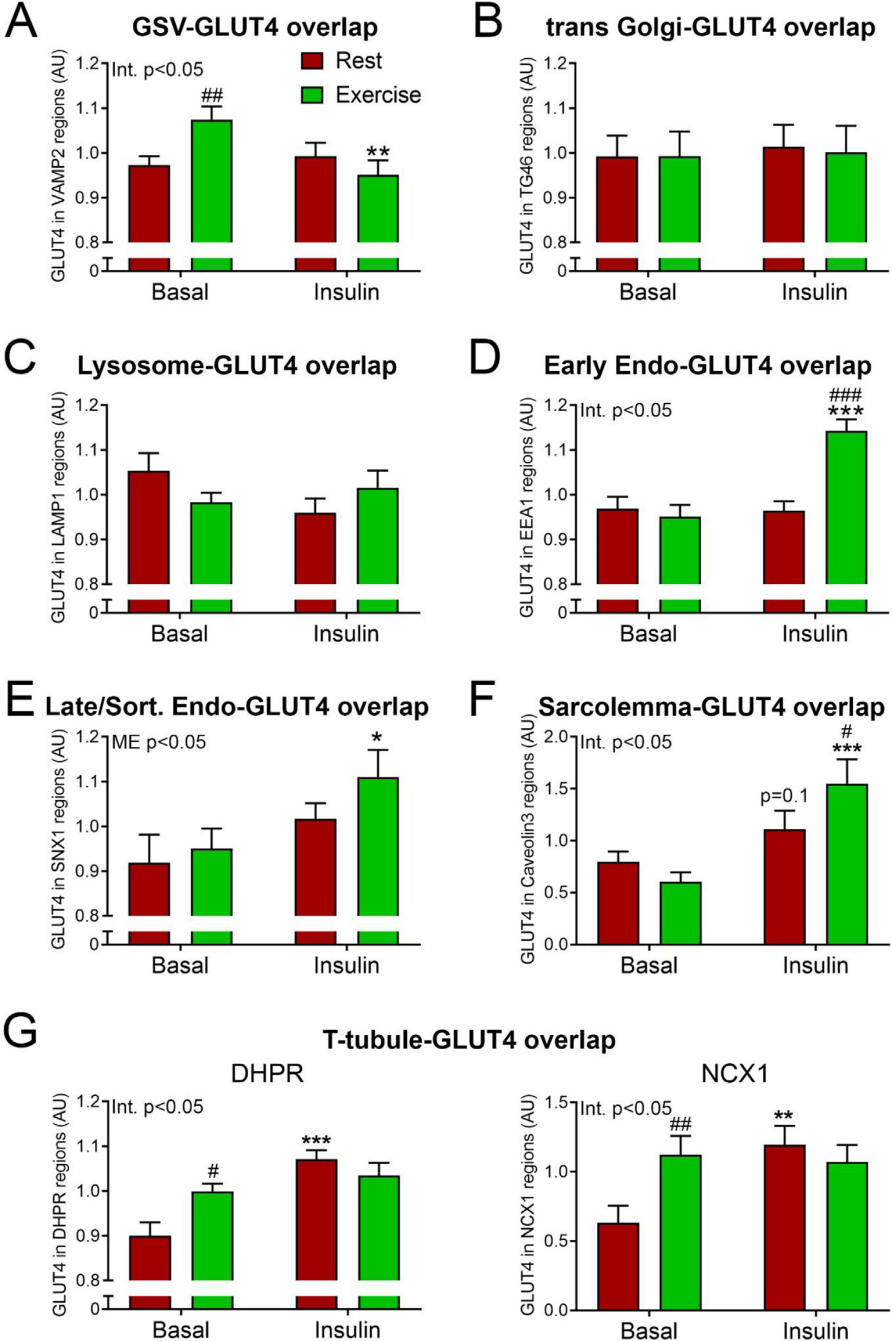
**Prior exercise and insulin redistributed intramyocellular GLUT4 in humans.** Human muscle biopsies from resting and prior exercised leg before and after insulin stimulation were cut in 70 nm sections at cryogenic temperature. Sections were labelled with GLUT4 and various compartment markers, and the GLUT4 content in each compartment was estimated using Mander's overlap coefficient. (A) GLUT4 overlap with vesicle associated membrane protein 2 (VAMP2) in human skeletal muscle. (B) GLUT4 overlap with trans Golgi network protein 2 (TGN46). (C) GLUT4 overlap with lysosome associated membrane protein 1 (LAMP1). (D) GLUT4 overlap with early endosome antigen 1 (EEA1). (E) GLUT4 overlap with sorting nexin 1 (SNX1). (F) GLUT4 overlap with caveolin 3 (Cav3). (G) GLUT4 overlap with dihydropyridine receptor α1 (DHPR) and Sodium–Calcium Exchanger 1 (NCX1). GSV = GLUT4 Storage Vesicles, Early Endo = Early endosomes, Late/Rec. Endo = Late/Recycling endosomes. n= (basal rest; basal exercise; insulin rest; insulin exercise) A)41; 38; 40; 29, B)32; 31; 31; 24, C)25; 30; 29; 22, D)34; 27; 33; 25, E)19; 20; 25; 19, F)61; 52; 55; 49, G)37; 36; 35; 36(DHPR) and 25; 24; 23; 23(NCX1). #/##/###p < 0.05/0.01/0.001 different from resting leg. ∗/∗∗/∗∗∗p < 0.05/0.01/0.001 effect of insulin stimulation.

**Figure 3:**
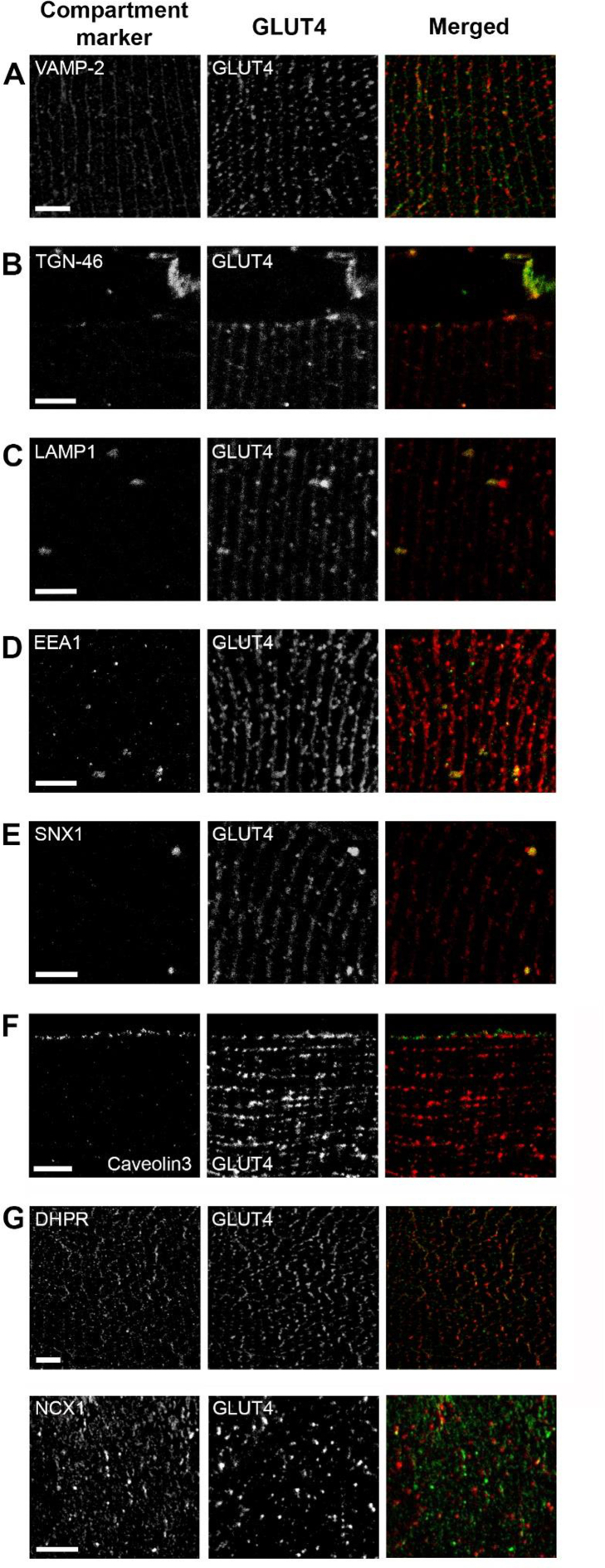
**GLUT4 positive compartments in human skeletal muscle.** Individual and merged images of GLUT4 and different compartment markers. Human muscle biopsies from resting and prior exercised leg before and after insulin stimulation were cut into 70 nm sections at cryogenic temperature. (A) GLUT4 and vesicle associated membrane protein 2 (VAMP2) antibody labelling in human skeletal muscle. (B) GLUT4 and trans Golgi network protein 2 (TGN46) antibody labelling. (C) GLUT4 and lysosome associated membrane protein 1 (LAMP1) antibody labelling. (D) GLUT4 and early endosome antigen 1 (EEA1) antibody labelling. (E) GLUT4 and sorting nexin 1 (SNX1) antibody labelling. (F) GLUT4 and caveolin 3 labelling. (G) GLUT4 and dihydropyridine receptor α1 (DHPR) and Sodium–Calcium Exchanger 1 (NCX1) antibody labelling. Scale bar = 5 μm.

In the current study, we needed to assign GLUT4 to different subcellular membrane compartments with high resolution and confidence in human skeletal muscle biopsies. This task is challenging, since GLUT4 is abundantly expressed in skeletal muscle and known to reside throughout the endomembrane system, including small vesicular structures, presumably making existing workflows with low Z-plane resolution and use of detergents unsuitable. To overcome these issues, we wondered if simply cutting the sample thinner would allow us to improve Z-resolution and avoid the use of detergents. Therefore we used the classical Tokuyasu ultracryotomy technique [18] to prepare ultra-thin muscle biopsy sections. A schematic overview of the workflow for the sample preparation is shown in Figure 1B. By infiltrating the muscle tissue with sucrose and embedding it in gelatin, we succeeded in cutting 70 nm sections from the biopsies. For reference, conventional muscle cryo-sections are ∼7 μm and isolated human muscle fibers vary in thickness from 40 to 80 μm [19]. Thus, this approach effectively improved the Z-plane resolution by almost 1 order of magnitude compared to the ∼600 nm resolution limit of confocal microscopy [20,21]. Furthermore, this sample-preparation technique, which we term Sample Thinning Enhanced Resolution Microscopy (STERM), also allowed antibody penetration in the absence of detergent (Figure 1C). The STERM method markedly improved the ability to resolve GLUT4 present in small vesicles from larger membrane structures (Figure 1B–C) using an otherwise standard work-flow for confocal microscopy. By visualizing GLUT4 in STERM-prepared human muscle samples using transmission electron microscopy, we confirmed GLUT4 to localize to cytosolic perinuclear, intramyofibrillar and subsarcolemmal areas (Figure 1, Figure 2, Figure 3), tubulovesicular structures (Figure 1D4), multivesicular endosomes (Figure 1D5) and, most critically, small vesicles sized ∼70–150 nm (Figure 1D5-6), some of which would presumably be detergent-sensitive. Thus the simple approach of combining Tokuyasu sections with confocal microscopy vastly improved overall image resolution of endogenous GLUT4 in human skeletal muscle without compromising antigenicity or using permeabilization reagents.

### 2.2 Prior exercise increases GLUT4 in insulin-responsive GSVs and T-tubuli

To pinpoint the localization of GLUT4 to specific subcellular compartments, we next co-labeled the STERM sections with GLUT4 and eight different compartment markers and quantified the amount of overlap between GLUT4 and the respective markers. In our analyses, we imaged several individual fibers in fixed biopsy material from three subjects. Because a between- and within-subject variation analysis showed that the variation within a subject was much larger than the subject-to-subject variation (Figure S1), we treated each image in our data analysis as a biological observation rather than a technical replicate. First, we examined the amount of GLUT4 in the specialized intracellular insulin responsive storage compartment, termed GLUT4 storage vesicles (GSVs). GLUT4 in GSVs is likely indicative of the capacity for insulin-responsive GLUT4 membrane insertion since insulin causes a reciprocal depletion of GLUT4 from these structures while increasing GLUT4 in the plasma membrane [22]. Interestingly, prior to insulin stimulation, we observed increased GLUT4 in the GSVs in the previously exercised leg (Figure 2A), measured as an overlap between GLUT4 and the well-described GSV marker, vesicle-associated membrane protein 2 (VAMP2) [23] (Figure 3A). There were no differences between the rested and the exercised leg in the overlap of GLUT4 and the trans-Golgi network (TGN) (Figure 2B) and the lysosomes (Figure 2C) marked by TGN-46 [24] (Figure 3B) and LAMP1 [25] (Figure 3C), respectively. Furthermore, we found no changes of GLUT4 content within the endosomes measured as GLUT4 overlap with the early endosomes (Figure 2D) and late/recycling endosomes (Figure 2E), identified by Early endosome antigen 1 [26] (Figure 3D) and Sorting Nexin 1 [27] (Figure 3E) prior to the insulin stimulation. When examining GLUT4 content at the surface membranes in the two legs before insulin stimulation, we observed the same amount of GLUT4 overlapping with the sarcolemma (Figure 2F) marked by Caveolin3 [28] (Figure 3F). However, we intriguingly detected an increased GLUT4 overlap with the T-tubules in the prior exercised leg before insulin stimulation (Figure 2G), using either the dihydropyridine receptor α1 (DHPR) [29] or the Sodium–Calcium Exchanger 1 (NCX1) [30,31] (Figure 3G). Thus prior exercise caused a marked intra-myocellular redistribution of GLUT4 4 h post-exercise preceding insulin-stimulation.

### 2.3 Prior exercise enhances the insulin-stimulated GLUT4 increase in the sarcolemma and endosomes

GLUT4 proteins originating from GSVs are inserted in the cell surface membranes upon insulin stimulation [23]. In this study, insulin stimulation resulted in an overall depletion of GLUT4-containing GSVs in the prior exercised leg (Figure 2A). No changes of GLUT4 content in GSVs were observed in the resting leg (Figure 2A). Remarkably, insulin stimulation at the same time led to an increased amount of GLUT4 in the early endosomes (Figure 2D) and the sarcolemma (Figure 2F) in the prior exercised leg compared to the resting leg. We observed no effect of insulin-stimulation on GLUT4 content in the TGN or lysosomes (Figure 2B–C), while insulin led to an increased amount of GLUT4 in the late/recycling endosomes in both legs (Figure 2E) and increased the T-tubular GLUT4 content in the resting leg to reach the level of the prior exercised leg (Figure 2G). Thus prior exercise appears to sensitize the insulin-stimulated redistribution of GLUT4 from GSVs to the sarcolemma and early endosomes in humans.

## 3 Discussion

The current data provide the first direct evidence in any species that a single insulin-sensitizing exercise bout redistributes intramyocellular GLUT4 to insulin-responsive GSVs and T-tubuli. Furthermore, we demonstrate that exercise augments skeletal muscle insulin-stimulated GLUT4 translocation to sarcolemma and endosomes post-exercise in humans, providing an important translational validation of previous observations in rodent muscle. These data strongly suggest that intramyocellular redistribution of GLUT4 contributes to the insulin-sensitization of skeletal muscle glucose uptake post-exercise. Together with previous studies showing enhanced insulin-stimulated muscle blood flow and microvascular perfusion [13], muscle surface membrane permeability [32], and increased glycogen synthase activity in humans 3–4 h post-exercise [5,8] it has now been demonstrated in humans that prior exercise causes a coordinated increase in insulin-stimulated glucose delivery, transport capacity and storage [14].

We presently observed a striking increase in co-localization of GLUT4 and VAMP2 4 h post-exercise and insulin-stimulated depletion of this co-localization in the prior exercised leg. VAMP2 is involved in the insulin-induced insertion of GLUT4 in the surface membrane but not maintenance of steady state membrane GLUT4, which undergoes constitutive recycling [33], whereas IRAP, the first identified co-occupant to GLUT4 in GSVs, co-localizes with GLUT4 in all of its intracellular compartments [34]. VAMP2 may therefore be the better marker of insulin-responsive GSVs. This suggests that prior exercise promotes biogenesis of insulin-responsive GSVs, allowing a greater pool of GSVs to be released upon insulin stimulation. Interestingly, a recent study showed that Sec16A bound to GTP-loaded Rab10 GTPase at the RE/TGN, thereby initiating a COPII coat protein-mediated GSV formation [35]. Rab10 signals downstream of TBC1D4 in 3T3-L1 adipocytes [36], presumably being controlled by the RabGAP activity of TBC1D4. Given that prior exercise modifies basal and insulin-stimulated TBC1D4 phosphorylation, this might contribute to enhanced GSV biogenesis. However, it should be noted that TBC1D4 regulation of GLUT4 translocation has been shown to involve Rab8a, 13 and 14 but notably not Rab10 in L6 rat myotubes [37,38].

Prior exercise also increased GLUT4 co-localization with the T-tubule markers DHPR and NCX1 prior to insulin-stimulation. Although the contribution of T-tubules to total muscle surface area varies with fiber-type and species, live-imaging of GLUT4 in mouse skeletal muscle has documented a role of this system in the insulin-induced GLUT4 movement [39]. Furthermore, crude disconnection of the T-tubular connection to the sarcolemma by osmotic shock in incubated mouse skeletal muscle reduced basal glucose uptake by 50% and prevented insulin-stimulated glucose uptake [40], suggesting the importance of T-tubuli for insulin-stimulated muscle glucose uptake. Since muscle glucose uptake is not enhanced 4 h post-exercise prior to insulin-stimulation [7,12,13], we suspect the T-tubular increase in GLUT4 to reflect a greater amount of pre-docked GLUT4 vesicles close to the T-tubuli rather than fully inserted GLUT4 vesicles [41]. Methodologically, we used confocal microscopy with a lateral resolution limit of ∼200 nm and an axial resolution of ∼70 nm. With a phospholipid membrane-bilayer thickness of ∼10 nm and GSV sizes of 50–70 nm, it is therefore impossible to distinguish GSVs close to the T-tubuli from GLUT4 inserted in T-tubuli. Another possible explanation would be that GLUT4 requires intrinsic activation by insulin, a concept suggested repeatedly [[42], [43], [44]] but not firmly established. In addition, it has been suggested that some types of exercise disrupt the T-tubule network [45]. If this is the case with the strenuous exercise protocol used here, then the increased T-tubular GLUT4 could represent non-functional GLUT4 in disrupted T-tubules inserted during contractions. Finally, glucose uptake 4 h post-exercise might be limited by a falling interstitial glucose concentration post-exercise [46], likely because blood flow and hence glucose delivery is non-significantly/mildly enhanced at this time point [13]. If correct, then increasing muscle blood flow artificially by, for example, infusing ATP, should be sufficient to enhance insulin-stimulated muscle glucose uptake. Regardless of these uncertainties, the increased T-tubular GLUT4 in the prior exercised leg would be predicted to augment insulin-stimulated T-tubular GLUT4 insertion and muscle glucose uptake. Surprisingly, there was no effect of prior exercise on insulin-stimulated T-tubular GLUT4 2 h into the clamp. A possible explanation is that we might have missed an insulin-sensitizing effect of prior exercise on T-tubular GLUT4 content by muscle sampling after 2 h of insulin-stimulation, where an initial effect of exercise during insulin-stimulation may have disappeared. This proposal is consistent with the consistently greater initial slope of increase in muscle glucose uptake and overall difference in the exercised vs. non-exercised leg observed during a clamp [7,12].

Unlike the T-tubuli, insulin-stimulated GLUT4 translocation was enhanced to the sarcolemma, EE and LE/RE compartments after 2 h of insulin-stimulation. Insulin is proposed to cause a dose-dependent release of GSVs into an endosomal recycling pool, which then recycles to the cell surface in the continued presence of insulin [47]. This model fits with our current observation of increased EE and LE/RE GLUT4 but no difference in TGN and strong depletion of GSVs. Thus prior exercise appears to increase the endosomal pool of GLUT4 recycling to the surface membrane, presumably by a greater initial depletion of GSVs. Consistent with these data, prior AICAR treatment of mouse EDL and soleus muscle fibers resulted in an increased insulin-stimulated GLUT4 translocation to endosomal compartments measured as GLUT4 particle size [48]. It is unclear if the T-tubular GLUT4 enters the endosomal recycling pool, either directly or via endocytosis from the cell surface, or is segregated from this membrane system. It is equally unclear to what extent the observed changes in GLUT4 translocation reflect changes in exocytosis and/or endocytosis rates.

AMPK is thus far the only exercise-stimulated protein demonstrated in mice to be required for insulin sensitization of muscle glucose uptake in mouse EDL after *in situ* contraction [49,50]. The primary candidate linking AMPK to insulin-sensitization is the Rab GAP protein TBC1D4 [51]. AMPK is required for TBC1D4 Ser711 phosphorylation and insulin-sensitization of muscle glucose uptake after *in situ* contraction in mice [49], and the corresponding site TBC1D4 Ser704 is also increased in humans at 3 h post-exercise [52]. Exactly how the AMPK-TBC1D4 signaling axis is required for muscle insulin sensitivity remains unknown. Future studies should aim to identify how exactly AMPK signals to increase insulin sensitivity and which GLUT4 pools are affected by AMPK stimulation and inhibition.

Although this study provides a valuable proof of the concept that GLUT4 redistribution post-exercise may be an underlying mechanism for increased post-exercise insulin sensitivity in humans, our data are still somewhat preliminary. Specifically, our study is based on biopsies from only three subjects. In addition, as mentioned above, our temporal resolution with one biopsy before and one 2 h into an insulin clamp is also poor. Thus our study needs to be followed up in other, preferably larger human study cohorts and at greater temporal resolution.

In summary, our study demonstrates that enhanced insulin-sensitivity in human skeletal muscle fibers post-exercise is associated with an intramyocellular redistribution of GLUT4 to GSVs and T-tubuli prior to insulin-stimulation. Furthermore, prior exercise augmented insulin-stimulated GLUT4 translocation to the sarcolemma and endosomal compartments. These data support a model of the post-exercise insulin sensitization phenomenon in which GLUT4 redistributes post-exercise into an insulin-responsive pool to allow greater GLUT4 mobilization to the cell surface and muscle glucose uptake by a given insulin-dose. This provides an explanation of why exercise serves as a cornerstone in the management of muscle insulin resistance.

## 4 Experimental procedures

Further information and requests for resources and reagents should be directed to and will be fulfilled by the Lead Contact, Thomas Elbenhardt Jensen (tejensen@nexs.ku.dk).

## 5 Experimental model and subject details

### 5.1 Human subjects

Muscle biopsies were obtained from three young healthy men as part of another study [53]. The study was approved by the Copenhagen Ethics Committee (H-6-2014-038) and complied with the ethical guidelines of the *declaration of Helsinki II*. Written informed consent was obtained from all subjects prior to enrollment in the study. The subjects were 28 ± 2 years old, with a BMI of 23 ± 1 kg m^−2^ and had a peak oxygen uptake (VO_2peak_) of 50 ± 5 ml min^−1^ kg^−1^.

### 5.2 Experimental protocol

The subjects underwent an incremental test to exhaustion on a Monarch ergometer cycle (Ergomedic 839 E, Sweden) to determine their VO_2peak_ by breath-by-breath measurements of VO_2_ (Masterscreen CPX, IntraMedic, Denmark) a minimum of one week prior to the experimental day. The subjects were also familiarized with the one-legged knee-extensor ergometer [54] and their peak work load (PWL) was determined for both legs. Subjects were instructed to abstain from alcohol and physical activity 48 h before the experimental day.

On the experimental day, the subjects were allowed to ingest a small breakfast (oatmeal, skimmed milk and sugar, corresponding to 5% of daily energy intake) before their arrival in the lab. After arrival, the subjects performed knee-extensor exercise for 1 h at 80% of PWL with 5 min intervals of 90% of PWL every 10 min. This was followed by 4 min fatigue bouts every 5 min until exhaustion. These started at 100% of PWL and declined 10 percentage points when subjects were not able to maintain a kicking frequency of 60 rpm. The kicking was terminated when the subjects were not able to end a fatigue bout at 60% of PW. The total kicking time was 131 ± 9 min. After exhaustion, the subjects rested in the supine position. Four hours later, a hyperinsulinemic-euglycemic clamp was initiated with a bolus of insulin (9 mU kg-1, Actrapid, Novo Nordisk, Denmark) followed by 120 min of constant insulin infusion (1.4mU min-1kg-1). A 20% glucose solution was used for glucose infusion and adjusted throughout the clamp to maintain euglycemia. Immediately before and after the clamp, muscle biopsies of *m. vastus lateralis* were obtained from both the resting and prior exercised leg using the Bergström needle technique with suction [55]. This protocol increased insulin-stimulated glucose uptake in the prior exercised leg compared to the rested leg [53].

### 5.3 Animals

C57BL6JRj female mice were used for optimization purposes related to Figure 1. The animal experiment was approved by the Danish Animal Experimental Inspectorate and complied with the European Union legislation, as outlined by the European Directive 2010/63/EU. The current work adhered to the standards outlined in the ARRIVE reporting guidelines. Mice were anesthetized by 2% isoflurane and a canula was then inserted into the left ventricle while cutting the right atrium open. Mice were then perfused with 0.1 M sodium phosphate buffer solution at pH 7.4 containing 4% paraformaldehyde (Electron Microscopy Sciences) and 0.05% glutaraldehyde (Sigma, G5882). Tibialis anterior muscles were excised and further kept in fixative for 4 h on ice before storage at 4 °C in phosphate buffer containing 1% paraformaldehyde.

## 6 Method details

### 6.1 Tissue preservation

Immediately after the biopsy procedure, a piece of the tissue was immersed in an ice-cooled 0.1 M sodium phosphate buffer solution at pH 7.4 containing 4% paraformaldehyde and 0.05% glutaraldehyde for fixation. Biopsy samples were kept on ice and finely divided into smaller bundles of <30 fibers before incubation on ice with shaking for 4 h. After fixation, the biopsies were stored in phosphate buffer containing 1% paraformaldehyde at 4 °C.

### 6.2 Sectioning and staining

Ultra-thin cryo sections were cut from tissue biopsies using the Tokuyasu technique [18]. Muscle fiber bundles were washed three times in 0.1 M phosphate buffer and placed in drops of 37 °C heated 12% gelatin in 0.1 M phosphate buffer. Samples were then placed on ice and ∼2–3 mm blocks were cut under a stereo microscope. Next, the blocks were infiltrated with 2.3 M sucrose under rotation overnight at 4 °C. The next day the blocks were mounted on cryo-pins (Jeveka) using 2.3 M sucrose and plunged into liquid nitrogen. For sectioning, the pinned blocks were placed in a Leica EM UC7 with a FC7 cryo attachment cooled down to −120 °C. Using a diamond trimming tool (Trim20, Diatome), a block was trimmed until the fiber bundle was reached and then an area of 300 × 400μm was trimmed. Using a diamond knife, 70 nm thick sections were cut, collected in a 1:1 mixture of 2.3 M sucrose and 2% Methyl cellulose and placed on 13 mm cover glasses (VWR) for light microscopy and on 300 mesh copper grids coated with pioloform and carbon (AGG2430C, Agar Scientific) for electron microscopy.

For antibody labelling, sections were placed upside down on a drop of phosphate buffered saline and incubated for 30 min at 37 °C. Grids were then quenched in 0.15% glycine in phosphate buffered saline for 2 × 5 min. Next, sections were incubated in blocking buffer containing 2% bovine serum albumin (Merck) and goat serum (Gibco) for 2 × 5 min before 2 h of incubation with antibodies at room temperature. After antibody labelling, the sections were washed 4 × 5 min in blocking buffer and then labelled with secondary antibodies for 1 h. Finally, the sections were washed in 1:20 diluted blocking buffer for 2 × 2 min and then in phosphate buffered saline for 3 × 5 min. For TGN-46 and GLUT4 co-labelling, sections were first labelled with a secondary anti-goat antibody and subsequently washed and labelled with a secondary anti-rabbit antibody before the final washing steps. For light microscopy, sections were then mounted on glass slides using ProLong gold. For transmission electron microscopy, sections were incubated in 1% glutaraldehyde in PBS for 5 min and washed in a stream of ddH_2_O for 2 × 4 minutes and 10 × 1 minutes. Sections were then contrasted by 3% (W/V) uranyl acetate at room temperature for 5 min followed by 5 min in 0.3% uranyl acetate in 1.8% methyl cellulose mixture on ice in the dark. Sections were then picked up by drying loops and excess stain was drained onto Whatman filter paper 1 before they were left to dry.

GLUT4 was labelled with a rabbit anti-GLUT4 antibody (PA5-23052, Invitrogen) and an Alexa 594 fluoronanogold goat anti-rabbit antibody (A-24923, Invitrogen) or a 10 nm gold anti-rabbit antibody (810.311, Aurion). Myocellular compartments were marked by the following antibodies: mouse anti-VAMP2 (MAB5136, R&D Systems Inc.), sheep anti-TGN46 (AHP500G, Bio-Rad laboratories), mouse anti-LAMP1 (H4A3, sc-20011, Santa Cruz), mouse anti-EEA1 (Clone 14/EEA1, BD Biosciences), mouse anti-SNX1 (Clone 51/SNX1, 611,482, BD Biosciences), mouse anti-DHPRα1 (MA3-920, Invitrogen), mouse anti-NCX1 (GTX22869, GeneTex) and mouse anti-caveolin-3 (sc-5310, Santa Cruz). TGN46 were labelled with an Alexa 488 donkey anti-goat antibody (A-110555, Invitrogen) and the rest were labelled with an Alexa 488 fluoronanogold goat anti-mouse antibody (A-24920, Invitrogen).

### 6.3 Imaging

All fluorescence microscopy was performed with a Leica SP5 inverted system using a 63x, 1.4 oil blue lens. Alexa 488 and 594 fluoronanogold antibodies were excited with a 100 mW argon laser (488 nm) and a 10 mW solid state laser (561 nm), and emitted light was detected using PMT detectors with the emission filters set at λ_em_ 495–580 nm and 600–730 nm. Images were collected as 8-bit images. Transmission electron microscopy was performed using a Tecnai12 120 kV BioTwin Spirit transmission electron microscope (FEI) equipped with a bottom-mount Eagle CCD camera.

### 6.4 Quantification and statistical analysis

Data are shown as means ± SEM. Obtained images were imported into Fiji-ImageJ (National Institute of Health, USA), thresholding and co-localization was determined using the Coloc 2 function in the co-localization analysis tool. For image analysis, several sections from 12 to 20 muscle fibers from each condition were imaged, each image was counted as n = 1. All imaging and processing was performed blinded. The significance of statistical test differences was evaluated using 2-way ANOVA followed by Tukey's post hoc analysis as appropriate. In Figure 2F, the data failed equal variance testing and were therefore square root transformed. The p-value for statistical significance was set as p < 0.05.

## Author contributions

TEJ, JRK and PV conceived the study. DES, JRH, JRK, CHO, ZL, BK, EAR and JFPW performed the human experiments. JRK and LH performed the microscopy and image analysis. TEJ and JRK wrote the manuscript and all co-authors commented on the draft and approved the final version. TEJ is the guarantor of this work, has full access to all the data in the study and takes responsibility for the integrity of the data and the accuracy of the data analyses.

## Appendix A Supplementary data

Multimedia component 1 Figure S1. **Within- and between-subject variation of GSV-GLUT4 overlap.** GLUT4 overlap with vesicle associated membrane protein 2 (VAMP2) in human skeletal muscle post-exercise prior to the insulin clamp. Data points of the same color (blue, red, green) stem from the same subject. GSV = GLUT4 Storage Vesicles. ∗∗ T-test difference (p = 0.005) in the prior contracted leg vs. resting leg using all data-points (n = 38–41). Multimedia component 1
