# Supplementary figures and images for: Prior exercise in humans redistributes intramuscular GLUT4 and enhances insulin-stimulated sarcolemmal and endosomal GLUT4 translocation
Source: Mol Metab. 2020 Apr 17;39:100998. doi: 10.1016/j.molmet.2020.100998 (PMC7240215; doi:10.1016/j.molmet.2020.100998)

**
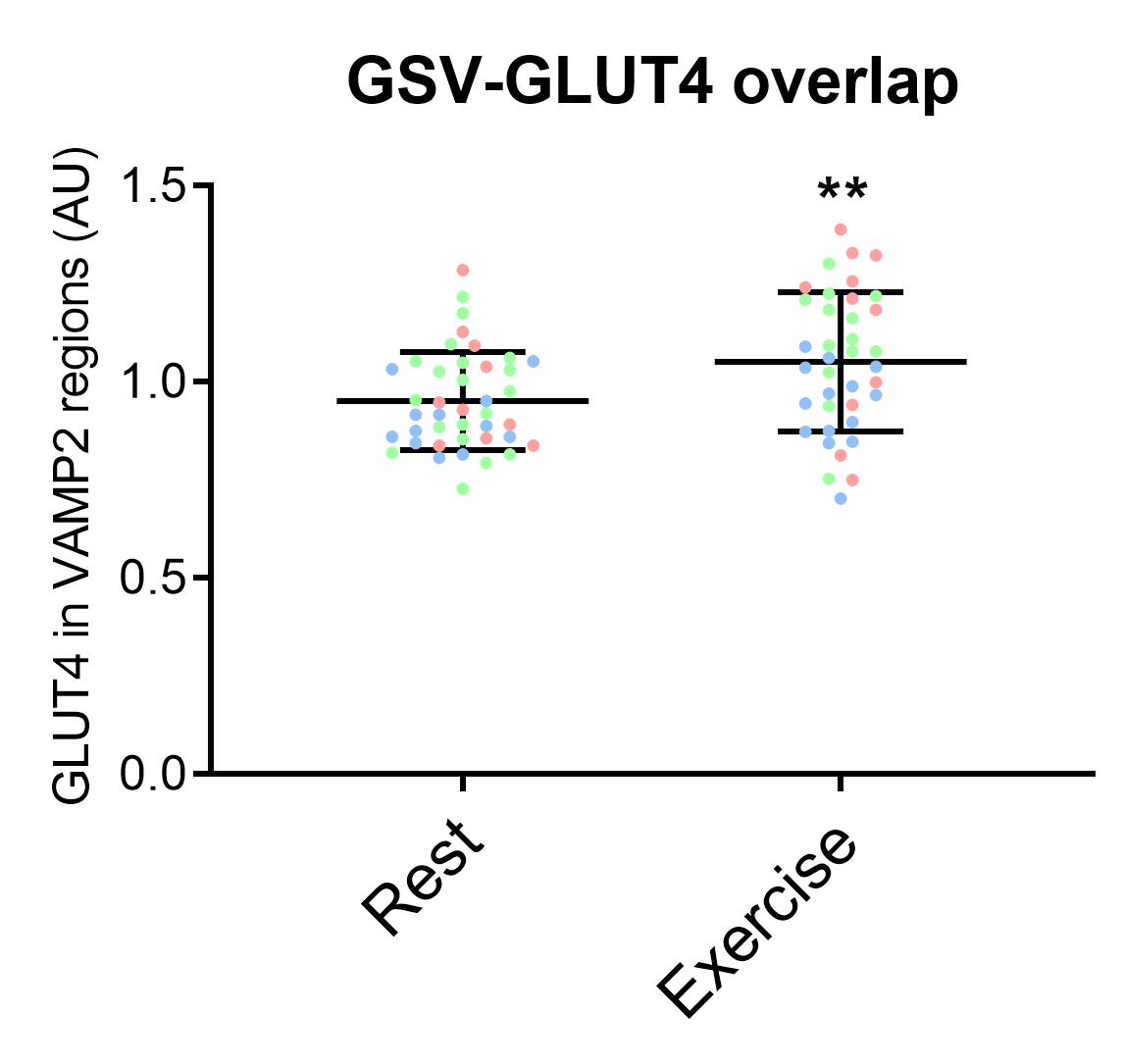
Figure S1.**

Supplement: Multimedia component 1 — Figure S1. Within- and between-subject variation of GSV-GLUT4 overlap. GLUT4 overlap with vesicle associated membrane protein 2 (VAMP2) in human skeletal muscle post-exercise prior to the insulin clamp. Data points of the same color (blue, red, green) stem from the same subject. GSV = GLUT4 Storage Vesicles. ∗∗ T-test difference (p = 0.005) in the prior contracted leg vs. resting leg using all data-points (n = 38–41). [file mmc1.docx]
